# MDM2 antagonists boost antitumor effect of androgen withdrawal: implications for therapy of prostate cancer

**DOI:** 10.1186/1476-4598-10-49

**Published:** 2011-05-03

**Authors:** Christian Tovar, Brian Higgins, Kenneth Kolinsky, Mingxuan Xia, Kathryn Packman, David C Heimbrook, Lyubomir T Vassilev

**Affiliations:** 1 Discovery Oncology, Roche Research Center, Hoffmann-La Roche Inc., Nutley, NJ 07110, USA

## Abstract

**Background:**

Hormone therapy is the standard of care for newly diagnosed or recurrent prostate cancers. It uses anti-androgen agents, castration, or both to eliminate cancer promoting effect of testicular androgen. The p53 tumor suppressor controls a major pathway that can block cell proliferation or induce apoptosis in response to diverse forms of oncogenic stress. Activation of the p53 pathway in cancer cells expressing wild-type p53 has been proposed as a novel therapeutic strategy and recently developed MDM2 antagonists, the nutlins, have validated this in preclinical models of cancer. The crosstalk between p53 and androgen receptor (AR) signaling suggest that p53 activation could augment antitumor outcome of androgen ablation in prostate cancer. Here, we test this hypothesis *in vitro *and *in vivo *using the MDM2 antagonist, nutlin-3 and the p53 wild-type prostate cancer cell line, LNCaP.

**Results:**

Using charcoal-stripped serum as a cellular model of androgen deprivation, we show an increased apoptotic effect of p53 activation by nutlin-3a in the androgen-dependent LNCaP cells and to a lesser extent in androgen-independent but responsive 22Rv1 cell line. This effect is due, at least in part, to an enhanced downregulation of AR expression by activated p53. In vivo, androgen deprivation followed by two weeks of nutlin administration in LNCaP-bearing nude mice led to a greater tumor regression and dramatically increased survival.

**Conclusions:**

Since majority of prostate tumors express wild-type p53, its activation by MDM2 antagonists in combination with androgen depletion may offer an efficacious new approach to prostate cancer therapy.

## Background

Despite advances in diagnostics and treatment, prostate cancer remains the second leading cause of cancer deaths in the US. Current treatments attempt to block cancer cell growth and induce cell death by removing or inhibiting the androgens that support tumor growth [1]. Surgical (orchiectomy) or chemical (LHRH agonist/antagonist) castration to eliminate testicular- androgen can delay clinical progression [2]. Anti-androgens such as flutamide or the more potent bicalutamide, which block the hormone-receptor interaction, have also been shown to improve survival [3-5]. Combined androgen blockade (CAB) applies both castration and anti-androgens, or estrogens to maximize the block on androgens including those produced from the adrenal gland. However, survival benefit from CAB is rather controversial and still under scrutiny [1]. Unfortunately, the majority of prostate cancer patients will eventually become resistant to one or all of these therapeutic strategies.

The mechanisms behind the resistance to androgen deprivation are not well understood although existing experimental evidence suggest that androgen withdrawal predominantly induces a cessation of cell proliferation but not overt apoptosis. In vitro studies with LNCaP cells grown in charcoal-stripped serum to mimic androgen ablation show a decrease in proliferation without apoptosis [6]. This is unlikely due to ineffective androgen removal because a recent study has indicated that tissue culture media supplemented with 10% fetal calf serum (FCS) contain castrate levels of testosterone and the level of androgen is well below serum levels of castrated males [7]. Normal rat prostate (and likely normal human prostate gland) respond to androgen ablation with high levels of apoptosis leading to glandular involution [8-10]. However, in human prostate cancer cells, the apoptotic response to androgen deprivation is not as clearly evident. It has been shown that androgen deprivation induces cell cycle arrest rather than apoptosis in three well known androgen-dependent cell lines, LNCaP, CWR22, and LuCaP-35 in vitro and in vivo [6,11,12]. Eventually, cell proliferation resumes, leading to an androgen-independent state in these model systems in vivo. This makes them a good model to assess the ability of therapeutics to induce cell death in combination with androgen ablation. The molecular response to in vivo androgen withdrawal was studied closely in the human prostate cancer xenograft model CWR22 in nude mice. Androgen ablation induced a robust stress response with an apparent p53-mediated cell cycle arrest but no p53-dependent apoptosis. Additionally the increased expression of p53 was only transient [11,13]. Lastly, studies of human tumor samples taken from patients that have undergone androgen deprivation show significant decreases in proliferation but minimal apoptotic index [9,10,14].

The p53 protein is a potent tumor suppressor that can induce cell cycle arrest or apoptosis in response to various forms of cellular stress [15]. Under non-stressed conditions, p53 is tightly controlled by its negative regulator MDM2 via an autoregulatory feedback loop [16,17]. p53 activates the transcription of the *mdm2 *gene and in turn MDM2 protein inhibits p53 transcriptional activity. In addition, MDM2 is a p53-specific E3 ligase which targets p53 for ubiquitination and degradation in the proteasome [18]. As a result of proper functioning of this autoregulatory loop both p53 and MDM2 are kept at low levels. In response to stress, the cellular levels of p53 increase leading to activation of multiple target genes and the p53 pathway with its main functions: cell cycle arrest and apoptosis [15,19]. These antitumor consequences make p53 a desirable target for pharmacological activation [20].

In addition to its role in cell cycle arrest and apoptosis, p53 has also been implicated in the regulation of AR [21]. Although the mechanism by which p53 exerts its control over AR is not clearly understood, p53 over-expression has been shown to decrease androgen function apparently by reduction in the expression of androgen-dependent genes [22,23]. However, this regulation is quite complex given that at physiological levels p53 may act to protect androgen signaling [21]. Conversely, androgen signaling has been found attenuated in etoposide-treated LNCaP cells as the stabilized p53 binds to the AR gene promoter [24]. Hence, p53 could facilitate the reduction of AR signaling by occupying and competing for AR promoter. A recent study has also implicated p53 negative regulator MDM2 in modulation of AR protein levels by targeting it for ubiquitin-dependent degradation [25].

We have demonstrated that a potent and selective small-molecule inhibitor of the p53-MDM2 binding, nutlin-3a, can stabilize p53 and activate the p53 pathway in a broad panel of wild-type tumor lines including prostate [26,27]. By disrupting p53-MDM2 regulatory circuit, nutlin elevates not only p53 but also MDM2, a transcription target of p53. Although MDM2 is kept away from p53, nutlin-bound MDM2 retains its E3 ligase activity against itself and possibly other targets. It has been documented that elevated MDM2 can facilitate MDMX ubiquitination and degradation in the presence of nutlin [28]. Therefore, one can expect that nutlin-induced elevated MDM2 could also facilitate AR degradation and further reduce AR levels when combined with androgen ablation. Here, we show that activation of the p53 pathway by nutlin-3a substantially augments the antitumor effect of androgen ablation. In vitro, combination of nutlin-3a with androgen deprivation in prostate cancer cell lines expressing wild-type p53 led to an increase in apoptosis over single agent treatments. More significantly, combination treatment of mice bearing LNCaP xenografts resulted in frequent complete tumor regressions and a dramatic increase in lifespan.

## Results

### Nutlin-3a activates the p53 pathway in LNCaP prostate cancer cells

**Figure 1:**
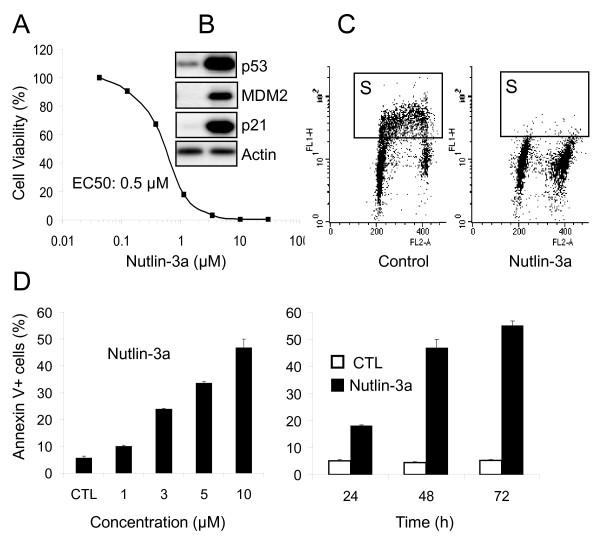
**Nutlin-3a activates p53 signaling in LNCaP cells**. A) Antitumor activity of Nutlin-3a. Cells were incubated with nutlin-3a for 5 days and cell growth/viability measured by the MTT assay. B) Changes in key proteins. Exponentially proliferating LnCAP cells were treated with 10 μM Nutlin-3a, or DMSO for 20 h and cell lysates were analyzed by Western blotting. C) Nutlin inhibits cell cycle progression. LNCaP cells were treated with 10 μM nutlin-3a for 20 h and analyzed by BrdU labeling and flow cytometry. Box indicates the S phase compartments. D) Nutlin induces dose and time-dependent apoptosis in LNCaP cells. Cells were incubated with nutlin-3a at the indicated concentrations and the percentage of the Annexin V-positive cells was determined. Time-dependant apoptosis was determined at 10 μM concentration.

To study the combined effect of p53 activation and androgen deprivation, we chose the androgen-depenedent prostate cancer cell line LNCaP as a cellular model. We have shown previously that nutlin-3a activates p53 and inhibits the growth of LNCaP cells and xenograft tumors in nude mice [27]. Incubation of proliferating LNCaP cells with nutlin-3a for 5 days showed a dose-dependent effect with EC_50 _of 0.5 μM (Figure 1A). Western blot analysis following 24 h nutlin treatment revealed p53 accumulation and concurrent increases in its transcriptional targets MDM2 and p21^Waf1/CIP1 ^(Figure 1B). Additionally, BrdU cell cycle analysis showed a loss of S-phase and increase of G1 and G2/M phase fractions consistent with p53-mediated cell cycle block in G1 and G2 phases (Figure 1C). To assess the apoptotic activity of nutlin-3a we used the Annexin V assay. Nutlin showed both a dose-dependant and time-dependant increase in the apoptotic fraction (Figure 1D). These results confirm that MDM2 antagonists stabilize p53 protein in LNCaP cells and effectively activate p53 signaling and the main p53 functions, cell cycle arrest and apoptosis.

### Nutlin treatment in combination with androgen deprivation further reduces AR levels and enhances apoptosis

**Figure 2:**
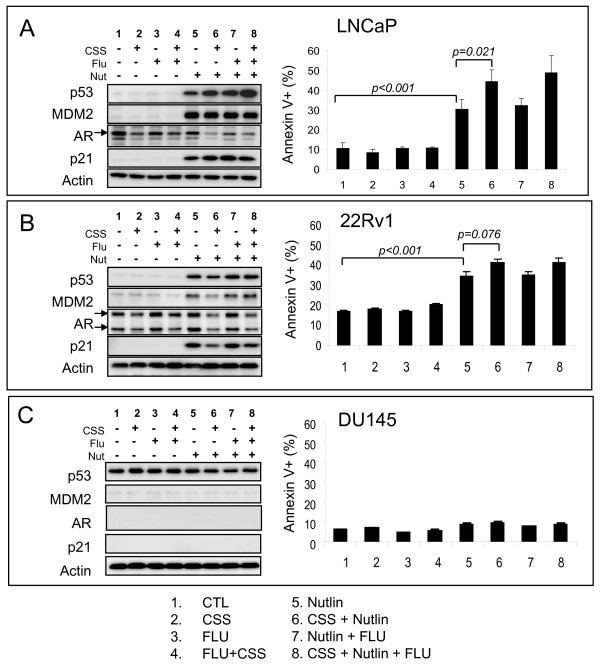
**Combination effect of nutlin and androgen deprivation in vitro**. LNCaP (A), 22Rv1 (B), and DU145 cells (C) were exposed to 5 μM Flutamide (FLU) and/or 5 μM Nutlin-3a in the presence of complete serum or charcoal stripped serum (CSS). Western blotting was used to monitor protein levels of Androgen receptor (AR) and the Annexin V assay to measure apoptotic response ± SD.

In addition to the androgen-dependent LNCaP cells, two other prostate cancer cell lines were chosen to investigate the combination effect of androgen ablation and p53 activation: 22Rv1 and DU145. Wild-type p53 expressing 22Rv1 cells have an androgen responsive AR but can grow independent of androgen in vivo. DU145 cells that express mutant p53 were used as a negative control since MDM2 antagonists require wild-type p53 for their cellular activity [26]. Cells were incubated in media containing either normal serum or charcoal-stripped serum (CSS) which has been shown to effectively eliminate androgen and thus mimic androgen ablation [7]. A suboptimal nutlin-3a dose of 5 μM was used (Figure 1D). Cells were also treated with 5 μM of the anti-androgen hydroxy-flutamide (FLU) in the presence of complete or CSS. Western blot analysis of LNCaP cells revealed that CSS in combination with nutlin elicits further reduction of AR levels than CSS alone in LNCaP cells and to a lesser extent in 22Rv1 cells (Figure 2A-B). Both LNCaP and 22Rv1 expressed full- length AR (110 kDa and 114 kDa respectively), 22Rv1 also expressed a 80 kDa isoform. Analysis of the AR mRNA levels in LNCaP cells by quantitative PCR revealed a 2-fold decrease in the presence of nutlin or CSS but no significant change in the combination (data not shown). Stabilization of p53 and subsequent increases in p21 and MDM2 levels were only observed in the presence of nutlin but not CSS indicating that CSS does not cause stress-related p53 activation.

Then, we examined the apoptotic response after 48 hour drug treatment (Figure 2). While CSS did not induce apoptosis over the control level in LNCaP cells, the combination with nutlin enhanced the apoptosis observed with nutlin alone (Figure 2A). Flutamide did not show a significant change in apoptosis. However, the triple combination (Nutlin + CSS + FLU) further increased the apoptotic index in LNCaP but not in 22Rv1 cells (Figure 2B). As expected, the p53-mutant and AR-negative line DU145 did not show an increase in p21 and MDM2. AR levels were also undetectable and no significant change in apoptotic fractions observed (Figure 2C).

### Bicalutamide (Casodex) enhances apoptotic activity of Nutlin-3a in LNCaP and 22Rv1 cells

**Figure 3:**
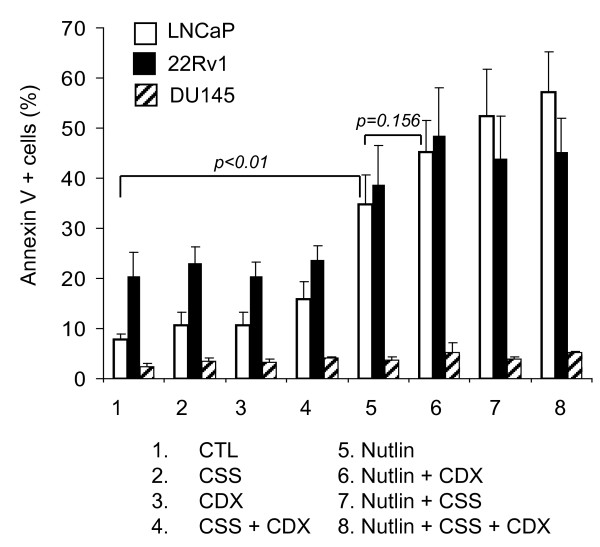
**Combination of Nutlin-3a and the anti-androgen casodex**. Exponentially growing LNCaP, 22Rv1, and DU145 were treated with 10 μM casodex (CDX) and/or 5 μM Nutlin-3a in the presence of complete serum or CSS and apoptotic fraction determined by the Annexin V assay ± SD.

Exponentially growing LNCaP, 22Rv1, and DU145 were incubated with 10 μM of another clinically used anti-androgen, Casodex (CDX), and/or 5 μM Nutlin-3a in the presence of complete serum or CSS. Percent apoptosis was determined by Annexin V staining after 48 hours (Figure 3). Data revealed that combined treatment with Nutlin-3a and CDX was more effective at inducing apoptosis than single agent treatments in both LNCaP and 22Rv1 cells. However, the combination of Nutlin-3a and CDX was less effective than Nutlin + CSS combination in LNCaP cells while triple treatment showed slight improvement over Nutlin + CSS combination. Nutlin + CDX combination showed a slightly better activity than Nutlin + CSS in 22Rv1 and triple treatment did not demonstrate greater effect over dual in this cell line.

### AR reduction accounts for the increased apoptotic activity of nutlin-3a in CSS

**Figure 4:**
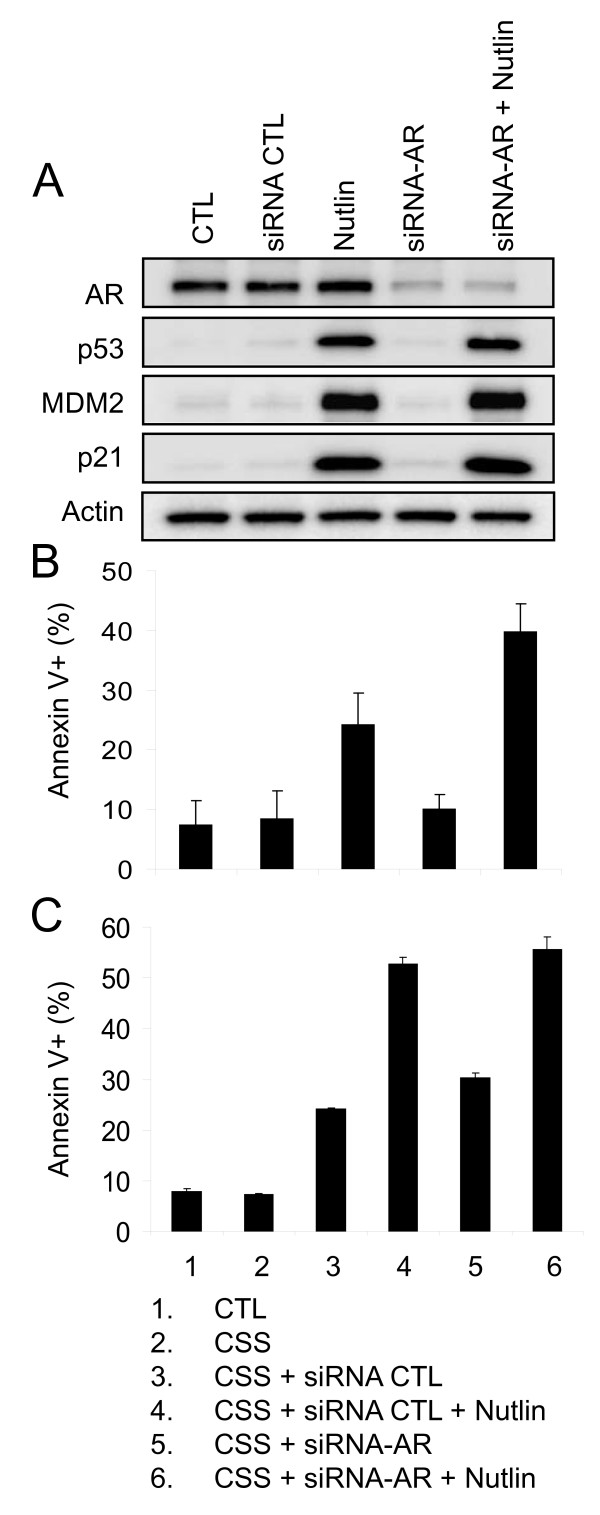
**Role of AR reduction in apoptotic response of LNCaP cells to nutlin**. A) Knockdown of AR in LNCaP cells by siRNA. siRNA (50 nM) was transfected in LNCaP cells and 24 h later 5μM Nutlin-3a was added for another 24 h before Western analysis B) AR knockdown increased apoptotic response to nutlin. Following 24 h knockdown of AR, LNCaP cells were washed and subsequently treated with 5 μM Nutlin-3a for a an additional 48 h and analyzed for Annexin V ± SD. C) AR knockdown does not increase further nutlin-induced apoptosis in CSS. LNCaP cells were transfected with siRNA for 24 h. Cells were then washed with PBS and maintained in CSS for an additional 48 h in the presence or absence of nutlin and apoptotic fraction measured as above.

To further investigate if the apoptosis enhancing effect of CSS is due to AR reduction, we depleted AR in LNCaP cells grown in normal serum using AR-specific siRNA. Western blotting confirmed the effective reduction of AR and showed that only in the presence of nutlin was there stabilization of p53 and activation of its transcription targets, p21 and MDM2 (Figure 4A). Addition of nutlin to AR-depleted LNCaP (siRNA-AR) cells led to a substantial increase in the apoptotic cell fraction over nutlin alone (Figure 4B). We then combined siRNA-AR and CSS with or without nutlin (Figure 4C). Apoptotic fraction of LNCaP cells in CSS + nutlin was nearly equivalent to the triple combination (CSS + siRNA-AR + nutlin), implying that CSS effectively reduces AR levels and that this is the main mechanistic cause for enhanced apoptotic response.

### Nutlin-induced AR reduction does not involve MDM2-dependant degradation

**Figure 5:**
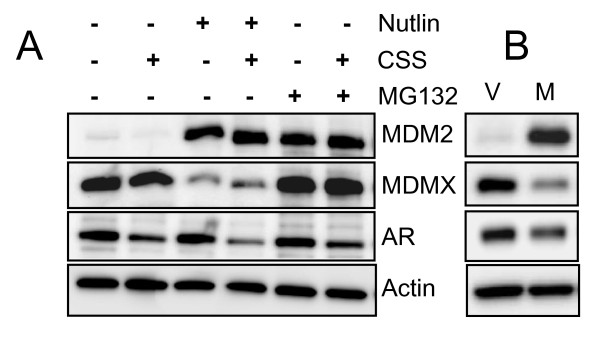
**Role of nutlin-induced MDM2 in AR degradation**. A) Nutlin-induced MDM2 facilitates the degradation of MDMX but not AR. LNCaP cells were grown in CSS in the presence or absence of 5 μM Nutlin-3a (24 h) and/or MG132 (8 h) and proteins were analyzed by Western blotting. B) LNCaP cell clone stably expressing high levels of MDM2 show a reduction in AR levels. Cell lysates from LNCaP cells transfected with a vector (V) and a clone stably expressing a full length MDM2 protein from an exogenous CMV-driven expression construct (M) were analyzed by Western blotting.

MDM2 has been shown to serve as an E3 ubiquitin ligase for p53 and several other proteins, most notably MDMX [28]. Moreover, AR degradation by Akt has been reported to require the E3 ligase activity of MDM2 [25]. Since nutlin treatment leads to a dramatic elevation of MDM2 protein one can expect facilitated degradation of AR, if nutlin-bound MDM2 can retain its activity against AR as previously shown with another MDM2 target, MDMX [28]. To test this possibility, we examined the protein levels of MDM2 and MDMX in the presence or absence of nutlin and/or CSS (Figure 5A). Nutlin treatment dramatically increased MDM2 levels and led to a significant reduction of MDMX but did not change AR protein levels. The reduction of MDMX was due to enhanced protein degradation since the proteasome inhibitor MG132 restored its levels. These results indicate that either MDM2 cannot facilitate AR degradation in LNCaP cells or that it has lost its activity against AR when in complex with the small-molecule inhibitor nutlin-3a. To distinguish between these possibilities, we generated a LNCaP cell clone stably expressing high levels of full length MDM2 protein under a CMV promoter. Western blot analysis showed significantly reduced levels of MDMX similar to nutlin-treatment. AR was also reduced but to a lesser extent. These experiments confirmed the previous observation that MDM2 protein can affect AR protein levels [25] and suggest that this ability is likely lost when nutlin-3a is bound to the MDM2 protein.

### Androgen deprivation does not affect nutlin-induced miR-34 expression

Recently, Rokhin at al. reported that LNCaP cells cultured in androgen-free media and then treated with the DNA-damaging agent, doxorubicin, suppressed the expression of p53 transcription targets miR-34a and miR-34c, resulting in inhibition of apoptosis [29]. It has been shown previously that nutlin activates miR-34(a-c) expression [30]. Therefore, we examined if the combination of nutlin and CSS can affect miR-34 levels. Exponentially proliferating LNCaP cells were incubated in CSS, 5 μM nutlin-3a, or combination of both for 24 h, and miR-34(a, b and c) levels were measured by quantitive PCR. We found significant induction of all three miRNA species by nutlin (34a:4.4, 34b:35.8, 34c:36.4 fold average) and less than 2 × fold increase in the presence of CSS. However, the combination did not significantly change the levels of any of the three miR over nutlin alone (data not shown). These experiments suggest that androgen deprivation does not affect miR-34 induction by non-genotoxic p53 activation.

### Nutlin-3a enhances antitumor activity of androgen deprivation *in vivo *and substantially increases lifespan of nude mice

**Figure 6:**
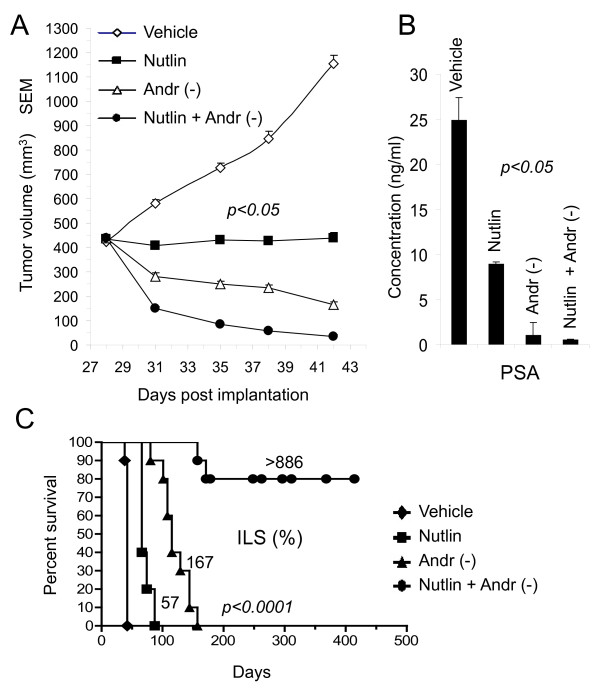
**Nutlin-3a greatly enhances antitumor activity of androgen deprivation in vivo**. A) LNCaP cells were grown subcutaneously in pre-castrated male athymic nude mice in the presence of testosterone pellets until reaching approximately 400 mm^3 ^on average. Mice were administered Nutlin-3a at 200 mg/kg bid (n = 10) or testosterone pellets were removed to simulate androgen ablation (n = 20). Additional mice were treated with a combination of ablation + Nutlin-3a at 200 mg/kg bid orally for 14 days (n = 20). Control mice were dosed with vehicle for 14 days + sham pellet removal under anesthesia (n = 10). B) PSA levels reflect changes in tumor volume. At day 42, five random mice in each group were bled and PSA levels ± SD were determined. C) Combination of nutlin and androgen deprivation increases lifespan of mice. After completion of treatment, mice were monitored for over a year and ILS was calculated by the Kaplan-Meier formula. Survival was calculated using a cut-off of 1000 mm^3^.

Finally, we examined if androgen ablation combined with nutlin-3a could enhance anti-tumor activity against established human prostate tumor xenografts (Figure 6A). To this end, pre-castrated nude mice were implanted with sustained-release testosterone pellets 5 days prior to injection of LNCaP cells. Tumors were fully established with an average starting tumor volume of approximately 400 mm^3 ^in each group. Mice were administered an optimal oral dose of nutlin-3a at 200 mg/kg twice a day (bid) for 14 days. Others were treated with removal of testosterone pellets as a model of androgen ablation. Additional mice were treated with a combination of ablation + nutlin-3a. Control mice were dosed with vehicle + sham pellet removal. Nutlin was well tolerated in all groups with no significant body weight loss in any group throughout the study. Tumor regressions were observed in all groups relative to vehicle. Nutlin-treated animals had 6/10 partial regressions, androgen ablation caused 20/20 partial regressions, and combination of nutlin + ablation yielded 13/20 partial regressions including 7/20 complete regressions (no complete regressions were observed in the other groups). At day 42 post tumor cell inoculation (last day of nutlin treatment), measurement of prostate specific androgen (PSA) in serum showed a 68% decrease with nutlin treatment alone compared to vehicle control (Figure 6B). Androgen ablation and combination treatment reduced serum PSA levels 94% and 98%, respectively.

Based on NCI criteria, an increase in lifespan (ILS) of ≥25% is biologically significant [31]. Twice daily dosing of nutlin-3a produced a 57% ILS and androgen deprivation gave a 167% ILS (Figure 6C). Although the difference in tumor volume at day 42 between androgen deprivation and combination treatment was only 6-fold (Figure 6A), over time, nutlin + androgen deprivation resulted in a greater than 886% ILS. This was the result of the majority of regressions never re-growing and the mice therefore died of perceived natural causes (no gross signs of primary subcutaneous tumor or metastasis upon necropsy in all cases). Collectively, our results demonstrate a clear benefit of administering nutlin-3a in conjunction with androgen ablation.

## Discussion

Although androgen withdrawal/deprivation is the predominant course of treatment for advanced prostate cancer, eventually all patients will develop resistance to this therapy. Moreover, the mechanism underlying hormone refractory disease progression has yet to be elucidated. Our *in vivo *data supports the conclusion that ablation elicits an impressive yet transient antitumor effect in concordance with early clinical response. However, akin to long term clinical results, our xenograft data shows that over time androgen deprivation does not result in sustained regressions (Figure 6C). Disappointingly, clinical results also show that most if not all advanced prostate cancers become androgen independent after surgical or chemical castration [1]. Taken together, these observations provide credence to the notion that ablation predominantly acts as an anti-proliferative rather than strong pro-apoptotic modality [11,14,32]. Further complicating matters is the apparent heterogeneity of advanced prostate tumors [33,34]. Taking this into account, initial anti-androgen therapy may simply eliminate a modest portion of a genetically-diverse androgen-dependant tumor cell population and provide selective pressure to the remaining malignancy that favors growth of new or pre-existing androgen-independent cell populations [35-37].

Although the requirement for androgen is no longer necessary in refractory disease the AR can still potentiate tumor growth and survival, thus activity of the AR remains a focal point of therapy even in advanced stages of androgen independent prostate cancer [38-40]. It is also evident that there are numerous mechanisms by which aberrant androgen signaling occurs in the development and progression of prostate cancer. Examples include AR hypersensitivity via gene amplification, changes in ligand specificity via mutation, increases in AR gene expression or androgen biosynthesis, all of which permit growth advantages in low androgen environments [37,40]. It is therefore imperative when using model systems to take into account the cellular milieu in which the AR exists. The LNCaP line used in this study for example, harbors the mutation T877A in the ligand binding domain. The consequence of this mutation is a loss of ligand specificity and promiscuous activation of AR with progestins, estrogens, adrenal androgens and surprisingly some anti-androgens.[37,38,41]. Consistent with published data, the androgen independent yet responsive 22Rv1 line show two mutant forms of the AR: a 114-kDa full-length form containing an exon-3 duplication and a truncated 80 kDa form which lacks the ligand binding domain ([42] and Figure 2B). In addition, the AR in 22Rv1 cells contains a His^874 ^to Tyr substitution that also permits stimulation by adrenal androgens albeit likely through a different mechanism than the T877A substitution in LNCaP cells [43]. Nevertheless, it is the AR that appears to be the principal accomplice in progressing towards complete androgen-independence [38-40].

Recent evidence suggests that the androgen receptor can confer cell survival by negatively regulating pro-apoptotic genes in the p53 pathway [41]. Moreover, overexpression of wild-type AR in LAPC4 or mutant AR in LNCaP lines can promote cell survival by inhibiting p53 mediated apoptosis [44]. Additionally, the potent androgen Dihydrotestosterone (DHT) has also been shown to decrease p53 levels and inhibit apoptosis in a dose dependent manner [45]. Alternatively, AR expression can be diminished by increased levels of p53 [24]. Highlighting the complex interplay between AR and p53 expression is the observation that physiological levels of p53 may be necessary to stabilize AR signaling [21]. Interestingly, LNCaP cells cultured in steroid-free media decreased both p53 protein and mRNA levels equal to that of treatment with high concentration of DHT. However, p53 knockdown in LNCaP cells have shown no change in AR levels regardless of DHT [45]. Taken together these findings suggest a mutual regulation of expression between p53 and AR. Therapeutic strategies aimed at disrupting the delicate cross-talk between AR and p53 must not only favor p53 expression, but also potentiate the apoptotic outcome of p53 activation.

Our published [27] and current data demonstrates that nutlin-3a alone has potent activity in the LNCaP cell line and in LNCaP xenograft models. As expected, nutlin-3a effectively activated the p53 pathway indicated by the increased expression of transcriptional targets, cell cycle arrest and apoptosis (Figure 1). These changes were not observed with CSS treatment and support the notion that androgen withdrawal by CSS does not cause stress-induced p53 activation. It is important to note that media supplemented with 10% fetal calf serum (FCS) does not represent normal physiological levels of testosterone but rather contain levels that are equivalent to that of serum in adult castrated males, nonetheless, LNCaP cells are able to maintain ~10 nM intracellular DHT concentration in 10% FCS by optimizing their AR and androgen metabolism [7]. Thus LNCaP cultured in CSS is exposed to testosterone levels significantly lower than that measured in serum from castrated adult males [7]. Unsurprisingly, we observed a discernible reduction in the expression of AR when LNCaP were cultured in CSS as opposed to FCS. Treatment with nutlin alone resulted in a relatively small decrease in AR protein levels (Figure 2B) likely reflecting the decrease of its mRNA levels. Greater reduction in AR protein occurred with concurrent nutlin and CSS treatment. This further reduction is not accounted for by the decrease in transcription or direct effect of elevated MDM2 on AR stability. This change may result from a complex set of events induced by activated p53 in an androgen-depleted milieu. Combination of nutlin and CSS enhaced the apoptotic response in LNCaP and to a lesser extent in 22Rv1 cells in vitro. (Figure 2). Presence of AR mutations in LNCaP and 22RV1 cells is likely responsible for the lack of robust in vitro response to flutamide as reported previously [38,41]. However, the second generation AR antagonist, casidex, did show clear in vitro effect. The decrease in AR protein levels appear to play a key role in the enhanced apoptotic response since siRNA-mediated knockdown of AR augmented apoptotic effect of nutlin similar to the combination with CSS. The mechanism behind the enhanced apoptosis in an AR-depleted environment is not entirely clear. One experimentally supported possibility is that AR activation attenuates p53-dependent apoptotic signaling [41,44] and efficient removal of AR expression leads to an overwhelming response in favor of cell death and not just cellular arrest.

*In vivo*, the combination of androgen ablation with nutlin treatment resulted in greater efficacy over corresponding monotherapy arms in established LNCaP xenografts and was the only group to show complete regressions in 7/20 mice despite the relatively high initial tumor volume (400 mm^3^). The greater degree of regression ultimately resulted in a dramatic increase of survival rates for the combination (Figure 6C). Taken together our data supports the notion that androgen withdrawal alone fails to induce a durable apoptotic response thereby allowing the eventual escape towards androgen independence.

## Conclusions

The administration of nutlin with androgen deprivation offers a novel two-pronged approach to cancer therapy in which factors that sustain prostate tumor growth are effectively removed with a simultaneous unleashing of the powerful growth suppressive and pro-apoptotic activity of p53. As increasingly more potent anti-androgens are identified (e.g. abiraterone acetate and MDV3100 [46]), their combination with MDM2 antagonists, currently in Phase I clinical development, could offer a compelling strategy in future trials.

## Methods

### Cells, reagents and treatment

All cell lines used in this study were purchased from the American Type Culture Collection (ATCC, Manassas, VA) and grown in the recommended media supplemented with 10% heat-inactivated FBS (Invitrogen, Carlsbad, CA) in a humidified environment with 5% CO_2_. Nutlin-3a was synthesized at Hoffmann-La Roche Inc, (Nutley, NJ). It was dissolved in DMSO and kept at -20°C as 10 mM stock solution. All other chemicals were purchased from Sigma (St. Louis, MO). Hydroxy-flutamide was dissolved in ethanol and maintained at 4°C at 10 mM stock concentration and used within 10 days. Bicalutamide was dissolved in DMSO and kept at 4°C as a 10 mM stock solution. MG132 was dissolved in DMSO and kept at -20°C in 10 mM aliquots. Cells were treated with 10 μM MG132 for 8 h prior to collection. For experiments in charcoal stripped serum (CSS) cells were washed with PBS and pre-incubated for 5 minutes in phenol free media with 10% CSS, washed again and replenished with phenol-free media containing 10% CSS with or without compound. The LNCaP-MDM2 overexpressing line was generated using the human full length MDM2 inserted into phCMV1 vector. The control is the empty phCMV1 and the selection marker is G418 at 250 μg/ml.

### Assays

Cell proliferation/viability was evaluated by the tetrazolium dye (MTT) assay [47] as previously described [26]. For cell cycle analysis, LNCaP cells (1 × 10^6 ^cells/T75 flask) were incubated with 10 μM nutlin-3a for 24 h. BrdU (20 μM; Sigma, St. Louis, MO) was added during the last 1 h prior to fixation and cells were processed and analyzed as previously described [27]. For apoptosis assays, cells were seeded in 6-well tissue culture plates at 2.5 × 10^5 ^(22Rv1 & DU145) or 5 × 10^5 ^(LNCaP) per well and treated for 48 h or as indicated. The percentage of Annexin V-positive cells was determined as described [27]. For qRT-PCR analysis, cells were seeded in 6-well plates, incubated overnight and treated with DMSO or 10 μM nutlin-3a for 24 h. Total RNA was isolated and analyzed as described previously [28]. To quantify microRNA expression, total RNA was isolated using the TRIzol solution (Invitrogen) following manufacturer's instruction. 10 ng of RNA was converted to cDNA using the TaqMan^® ^microRNA Transcription Kit and real-time PCR analysis was performed using TaqMan^® ^microRNA assays (Applied Biosystems). For Western blotting, cells were grown in 75 cm^2 ^flasks (10^6 ^cells per flask) or in duplicate in 6 well plates for siRNA experiments, lysed in 0.1 -0.2 ml RIPA buffer and analyzed as previously described [27]. For siRNA experiments, human-specific AR SMARTpool siRNAs and non-targeting control siRNA were obtained from Dharmacon RNAi Technologies (Lafayette, CO) and used as previously described [28].

### Animals Studies

Precastrated male athymic nude mice (Crl:NU-Foxn1nu) were obtained from Charles River Laboratories (Wilmington, DE) and cared for as previously described [27]. At 10 weeks of age, mice were implanted with a 12.5 mg sustained-release testosterone pellet (Innovative Research of America, Sarasota, FL) 5 days before the injection of tumor cells. A 1:1 mixture of human LNCaP prostate cancer cells suspended in phenol-free Matrigel and PBS were implanted in the right flank at a concentration of 1 × 10^7 ^cells in 0.2 ml total volume. The health of all animals was monitored daily by gross observation and analyses of blood samples of sentinel animals. All animal experiments were performed in accordance with protocols approved by the Institutional Animal Care and Use Committee in our AAALAC accredited facility.

Four weeks after inoculation, mice were assigned to different treatment groups with average tumor volume approximately 400 mm^3^. Mice were administered Nutlin-3a at 200 mg/kg bid (n = 10) orally for 14 days in 0.2 ml volume per injection or testosterone pellets were removed under ketamine/xylazine cocktail anesthesia to simulate androgen Ablation (AA) (n = 20). Other mice were treated with a combination of AA + Nutlin-3a at 200 mg/kg bid orally for 14 days (n = 20). Control mice were dosed with 0.2 ml vehicle bid orally for 14 days + sham pellet removal under ketamine/xylazine cocktail anesthesia. Tumor measurements and weights were taken 2-3 times per week. Tumor growth inhibition was calculated from percent change in mean tumor volume compared to the control group. Average percent weight change was used as a surrogate endpoint for tolerability in the experiment. Five random mice in each group were bled via the retrorbital sinus under anesthesia to obtain serum for PSA determination using a PSA ELISA (American Qualex, Inc., San Clemente, CA). Ten animals in each group were continuously followed beyond the last day of nutlin treatment to see if tumor re-growth would occur. In this second phase of analysis, survival was calculated using a cut-off individual tumor volume of 1000 mm^3 ^as a surrogate for mortality. The increase in lifespan (ILS) was calculated as % using the formula: [(Median day of death in treated tumor-bearing mice) - (median day of death in control tumor-bearing mice)] × 100 Median day of death in control tumor-bearing mice. Statistical analysis was performed as previously described [27].

## Competing interests

The authors declare that they have no competing interests.

## Authors' contributions

CT participated in the design and execution of most in vitro studies and drafted the manuscript. BH designed and carried out the in vitro studies, analyzed data and participated in the preparation of the manuscript. KK participated in the in vivo studies and data analysis. MX generated the LNCaP cell clones. KP participated in the in vivo study design and data analysis. DH participated in the drafting and critical revision of the manuscript. LV participated in study design and coordination, data analysis and interpretation and prepared the manuscript.

All authors have read and approved the final manuscript.
